# A Case–Control Study of Occupational Exposure to Trichloroethylene and Non-Hodgkin Lymphoma

**DOI:** 10.1289/ehp.1002106

**Published:** 2010-11-02

**Authors:** Mark P. Purdue, Berit Bakke, Patricia Stewart, Anneclaire J. De Roos, Maryjean Schenk, Charles F. Lynch, Leslie Bernstein, Lindsay M. Morton, James R. Cerhan, Richard K. Severson, Wendy Cozen, Scott Davis, Nathaniel Rothman, Patricia Hartge, Joanne S. Colt

**Affiliations:** 1 Division of Cancer Epidemiology and Genetics, National Cancer Institute, Rockville, Maryland, USA; 2 National Institute of Occupational Health, Oslo, Norway; 3 Stewart Exposure Assessments, LLC, Arlington, Virginia, USA; 4 Program in Epidemiology, Fred Hutchinson Cancer Research Center and Department of Epidemiology, University of Washington, Seattle, Washington, USA; 5 Department of Family Medicine and Public Health Sciences, Wayne State University, Detroit, Michigan, USA; 6 Department of Epidemiology, University of Iowa, Iowa City, Iowa, USA; 7 Division of Cancer Etiology, Department of Population Sciences, Beckman Research Institute, City of Hope, Duarte, California, USA; 8 Department of Health Sciences Research, Mayo Clinic College of Medicine, Rochester, Minnesota, USA; 9 Department of Preventive Medicine, University of Southern California, Los Angeles, California, USA

**Keywords:** cancer, non-Hodgkin lymphoma, occupational, solvents, trichloroethylene

## Abstract

**Background:**

Previous epidemiologic findings suggest an association between exposure to trichloroethylene (TCE), a chlorinated solvent primarily used for vapor degreasing of metal parts, and non-Hodgkin lymphoma (NHL).

**Objectives:**

We investigated the association between occupational TCE exposure and NHL within a population-based case–control study using detailed exposure assessment methods.

**Methods:**

Cases (*n* = 1,189; 76% participation rate) and controls (*n* = 982; 52% participation rate) provided information on their occupational histories and, for selected occupations, on possible workplace exposure to TCE using job-specific interview modules. An industrial hygienist assessed potential TCE exposure based on this information and a review of the TCE industrial hygiene literature. We computed odds ratios (ORs) and 95% confidence intervals (CIs) relating NHL and different metrics of estimated TCE exposure, categorized using tertiles among exposed controls, with unexposed subjects as the reference group.

**Results:**

We observed associations with NHL for the highest tertiles of estimated average weekly exposure (23 exposed cases; OR = 2.5; 95% CI, 1.1–6.1) and cumulative exposure (24 exposed cases; OR = 2.3; 95% CI, 1.0–5.0) to TCE. Tests for trend with these metrics surpassed or approached statistical significance (*p*-value for trend = 0.02 and 0.08, respectively); however, we did not observe dose–response relationships across the exposure levels. Overall, neither duration nor intensity of exposure was associated with NHL, although we observed an association with the lowest tertile of exposure duration (OR = 2.1; 95% CI, 1.0–4.7).

**Conclusions:**

Our findings offer additional support for an association between high levels of exposure to TCE and increased risk of NHL. However, we cannot rule out the possibility of confounding from other chlorinated solvents used for vapor degreasing and note that our exposure assessment methods have not been validated.

The International Agency for Research on Cancer (IARC) has evaluated trichloroethylene (TCE; C_2_HCl_3_) as a probable carcinogen (Group 2A), although the evidence of carcinogenicity in humans was noted as limited (IARC 1995). TCE has been a widely used chlorinated solvent since the early 1900s, primarily in industrial vapor degreasing operations for cleaning metal parts. Since the early 1970s, however, concerns over its environmental and health effects have led to a decline in TCE use and the increased use of other chlorinated solvents as vapor degreasing agents (Bakke et al. 2007). Other uses have been as a solvent in dry cleaning, food processing, textile scouring, and leather processing and in adhesives, drugs, paints, and other products (Bakke et al. 2007). TCE is also a common soil and water pollutant, released into the environment primarily through industrial wastewater and leaching from hazardous waste sites (Wu and Schaum 2000).

Concern that TCE may exert immunotoxic effects (Cooper et al. 2009) has motivated epidemiologic research investigating TCE exposure and risk of non-Hodgkin lymphoma (NHL), a malignancy linked to immune dysregulation. Occupational exposure to TCE has been associated with increased risk of NHL in several epidemiologic studies, although the overall published evidence is inconsistent (Mandel et al. 2006; Scott and Chiu 2006; Seidler et al. 2007; Wang et al. 2009; Wartenberg et al. 2000). This inconsistency may at least partly reflect limitations in the exposure assessment, because many of the previous studies, case–control studies in particular, inferred workplace exposure to TCE based on occupational title or through the use of job-exposure matrices (Scott and Chiu 2006). The underlying assumption with these methods—that workers in the same job or industry experience similar exposures to TCE—is questionable and may lead to substantial measurement error that, when independent of disease status and the magnitude of true exposure, typically produces bias toward the null (Armstrong 1998; Wacholder 1995; Wacholder et al. 1995). Additional evidence from large case–control and cohort studies that account for individual variability in exposure is needed to better understand whether TCE exposure is associated with increased NHL risk.

The National Cancer Institute–Surveillance, Epidemiology, and End-Results (NCI-SEER) study of NHL is a large population-based case–control study designed to obtain detailed information regarding workplace exposure to solvents through the use of job-specific interview modules (Gérin et al. 1985; Siemiatycki et al. 1981; Stewart et al. 1996).

This article reports findings from an analysis within NCI-SEER investigating the association between NHL and occupational TCE exposure, which was assessed by an expert industrial hygienist after a review of participants’ occupational histories and job-specific module data and of published exposure information.

## Materials and Methods

### Study population

The NCI-SEER case–control study of NHL has been described previously (Chatterjee et al. 2004; Schenk et al. 2009). Study participants were enrolled from four U.S. SEER registry areas: the State of Iowa; Los Angeles County, California; and the Seattle, Washington; and Detroit, Michigan, metropolitan areas. Eligible cases were individuals 20–74 years of age diagnosed between July 1998 and June 2000 with incident NHL according to the *International Classification of Diseases for Oncology* (Percy et al. 1990), without known HIV infection. Controls with no previous diagnosis of NHL were selected from the general population in the four registry areas by random digit dialing (RDD; < 65 years of age) and from residents listed in Medicare files (65–74 years of age), with stratification on the basis of age (5-year intervals), sex, race, and SEER area to match the distribution in the cases. The study was approved by the institutional review boards at the NCI and the participating institutions, and study participants provided informed consent.

Of the 2,248 potentially eligible cases, 320 (14%) died before they could be interviewed, 127 (6%) could not be found, 16 (1%) had moved away, and 57 (3%) had physician refusals. We attempted to contact the remaining 1,728; of these, 1,321 participated, which represented a participation rate of 76% and an overall response rate of 59%. Sixty-one percent of the cases were interviewed within 6 months after the diagnosis date, and 84% within 12 months after diagnosis.

Of the 2,409 potentially eligible controls identified from RDD (78% response rate) and Medicare files, 28 (1%) died before they could be interviewed, 311 (13%) could not be located, and 24 (1%) had moved away. We attempted to contact the other 2,046; of these, 1,057 participated, which yielded a participation rate of 52% and an overall response rate of 44%.

### Exposure assessment

Participants were mailed a residential and occupational history calendar. During a subsequent home visit, a trained interviewer administered a computer-assisted personal interview (CAPI), developed by our research team for this study, that covered a wide variety of topics, including an occupational history. The occupational history gathered information on each job held by the subject for 12 months or longer since the age of 16, including the name of the employer, dates of employment, job title, number of hours worked (full or part time), type of business or service, tasks, and materials and equipment used. In addition, for selected occupations, one of 32 job- or industry-specific interview modules was administered based on the information collected in the occupational histories. The modules focused specifically on solvent exposures, asking for detailed information over the entire duration of employment in a given job (e.g., machinist) or industry (e.g., dry cleaning industry). The information collected in the modules included the average frequency of various solvent-related tasks (converted to times per week), the average length of time it took to perform given solvent-related tasks (converted to hours per instance), sensory descriptions, dermal exposure, work practices, engineering controls, and personal protective equipment use (Gerin et al. 1985; Siemiatycki et al. 1981; Stewart et al. 1996). In particular, subjects who reported jobs that could involve degreasing work were asked the following information regarding degreasing: the usual number of hours per instance spent degreasing, whether they had ever used specific degreasing chemicals (including TCE), the percentage of time each chemical was used, whether the degreasing agent was at room temperature or heated, and the manner in which parts were cleaned (wiped with rag or cloth, wiped with brush, put in basket and dipped into tank, put into tank or bucket with hands).

Of the 1,321 cases and 1,057 controls that were interviewed, 132 cases (10%) and 75 (7.6%) controls were never employed or had unknown occupations. These subjects were excluded, leaving 1,189 cases and 982 controls for our analysis. The job modules were incorporated into the CAPI approximately 1 year into the interview phase of data collection; 682 cases and 640 controls were interviewed with a CAPI version that included these modules. The previously interviewed subjects were not recontacted. A maximum of five of the 32 job- or industry-specific modules was administered in an interview; six cases and four controls who reached the five-module limit had at least one additional job intended to trigger a module. All jobs were coded using the Standard Industrial Classification (SIC) and Standard Occupational Classification (SOC) systems ([Bibr b37-ehp-119-232][Bibr b38-ehp-119-232]).

A systematic review of the industrial hygiene literature for uses of TCE in U.S. industry provided important information for the exposure assessment (Bakke et al. 2007). Workplace, personal, and area TCE measurement data, TCE uses, and determinants of exposure (e.g., tasks, work practices) reported in the literature were collected, and the measurement data were then summarized by industry and “source of exposure” (degreasing, vapor degreasing, spot removal, printing dyes, etc.). Exposure matrices (*n* = 23), developed by the industrial hygienist using information from the literature review, provided initial estimates of probability, frequency, and intensity of exposure for different combinations of occupation, industry, and decade of employment.

Using the literature review, the exposure matrices, the occupational histories, and the information collected in the job modules, an expert industrial hygienist assessed levels of probability, frequency, and intensity of TCE exposure for each job. Probability, defined as the theoretical probability of exposure to TCE, was assigned to one of five categories: 0%, < 10%, 10–49%, 50–89%, or ≥ 90%. If the subject specifically reported TCE use, a probability of ≥ 90% was assigned. Otherwise, the probability was assigned by the industrial hygienist based on the likelihood of using TCE during the decade(s) the job was held and, for degreasing, at the temperature reported in the module (room temperature vs. heated). All jobs with a probability of > 0% were assigned an exposure frequency and intensity. Exposure frequency was assigned to one of four categories according to the estimated number of hours per week exposed to TCE: < 2, 2–9, 10–19, or ≥ 20 hr/week. The frequency assigned was either the reported frequency of performing the task or, if missing, the average frequency of all reports for that task. The exposure intensity was the average concentration of the solvent estimated by the industrial hygienist to have been in the subject’s breathing zone while exposed (i.e., while performing the task) in parts per million, and was assigned to one of five categories: < 1, 1–19, 20–99, 100–199, or ≥ 200 estimated ppm. Estimates of intensity were based primarily on previously published task-specific (i.e., “source of exposure” in Bakke et al. 2007) and decade-specific short-term personal measurements summarized in the literature review, as most tasks reported in the modules were short term. The intensity assignments were made irrespective of job title or industry; for example, mechanics and machinists who reported identical information on degreasing in the same time decades(s) were assigned the same exposure intensity scores. The intensity score also reflected dermal exposure. As mentioned above, one of the degreasing questions in the modules asked how parts were cleaned. If a subject indicated that he or she cleaned parts “with a rag” or “put [the parts] into a tank or bucket with hands,” the assigned intensity category was raised to the next higher category to reflect likely dermal exposure. Because the intensity scores represent estimates, are not based on direct monitoring data of the subjects’ work environment, and may reflect dermal exposure, we believe the parts per million estimates should be interpreted with caution. Lastly, we assigned an overall confidence score, which reflected the quality of the background information on which the estimates were based, on a three-point scale (low, medium, high). The exposure assessment was done without knowledge of the subject’s case or control status.

To support a systematic assessment of exposure, additional rules for anticipated exposure situations were developed a priori (e.g., if frequency of TCE exposure was < 15 min/week, the subject was assigned 0% probability of exposure with a low confidence score because exposure had occurred but probably not with a significant frequency). When the information collected during the interview was insufficient or when no module for a possibly solvent-exposed job was administered (mainly because the subject was interviewed before the modules were incorporated into the CAPI), the exposure matrices were used to estimate probability, frequency, and intensity of exposure. Information reported during the interview (e.g., a report of TCE use in the occupational history), however, always took precedence over the exposure matrices.

The job-specific estimates of probability, frequency, and intensity for each subject were integrated to develop several metrics of TCE exposure. We defined a subject as “unexposed” to TCE if all jobs had been assigned an exposure probability of 0%, “possibly exposed” if one or more jobs had been assigned an exposure probability of < 50%, and “probably exposed” if at least one job had been assigned an exposure probability of ≥ 50%. Subjects defined as “probably exposed” on the basis of degreasing work were so classified because they reported using TCE in the occupational history or a job module or reported in a job module performing heated degreasing during a time period when TCE was the vapor degreaser of choice and did not identify any other specific solvent.

For subjects defined as probably exposed, we calculated the following additional exposure metrics:

- Duration of exposure (years), defined as the sum of the number of years worked at each job across all jobs with exposure probability ≥ 50%.
- Cumulative exposure (estimated parts per million–hours), defined as the sum, across all jobs with exposure probability ≥ 50%, of the product of the job-specific intensity midpoint (0.25, 10, 60, 150, or 300 estimated ppm), the frequency midpoint (1, 6, 15, or 30 hr/week), and the duration in weeks.
- Average weekly exposure (estimated parts per million–hours per week), defined as the cumulative exposure divided by the duration of exposure in weeks.
- Average exposure intensity (estimated parts per million), defined as the duration-weighted average intensity level across all jobs with exposure probability ≥ 50%.

All of these metrics were set to 0 for unexposed subjects. Further adjustment of the exposure metrics using the exposure confidence score yielded virtually identical results and are not reported.

Other than the ever/never analysis, we did not include subjects who were possibly exposed in any analysis. This decision was made because the observed prevalence of possible TCE exposure among population-based controls in this study (42% of controls; Table 1) was unrealistically high given the narrow set of occupational applications for TCE (Bakke et al. 2007), thus suggesting poor specificity for this lower-stringency definition for exposure. Given these concerns regarding the expected specificity of this measure, and the importance of high specificity when evaluating rare exposures (Dosemeci and Stewart 1996), further analyses of this measure were judged as unlikely to be informative and thus not performed.

### Statistical analysis

All analyses were performed using the statistical software SAS (version 9.1.3; SAS Institute Inc., Cary, NC, USA), using α = 0.05 to indicate statistical significance. We described the associations between categories of each TCE exposure metric and NHL (with unexposed subjects as the referent) using odds ratios (ORs) and 95% confidence intervals (CIs) computed from unconditional logistic regression modeling with adjustment for age (< 45, 45–64, ≥ 65 years), sex, race (Caucasian, African American, other/unknown), education level (< 12, 12–15, ≥ 16 years), and SEER area (Detroit, Iowa, Los Angeles, Seattle). The exposure metrics were categorized using tertiles among probably exposed controls as cut-points. For analyses of average weekly exposure, years exposed, and cumulative exposure, we further subdivided the highest exposure category using the intracategory median among controls to investigate associations across a wider range of exposure levels. Tests for trend were performed by modeling the exposure metrics as continuous variables. In addition, ORs and 95% CIs from the continuous models were computed, using the difference between the second and third tertiles among exposed controls as the scale of reference for a given exposure metric.

We conducted analyses of specific histologically defined NHL subtypes (diffuse large B-cell lymphoma, follicular lymphoma, and small lymphocytic lymphoma and chronic lymphocytic leukemia) (Morton et al. 2007) using polytomous regression to explore possible heterogeneity in the association with TCE exposure. We also conducted analyses of all NHL, stratifying on sex, race, and age group (≤ 54, ≥ 55 years), and performed sensitivity analyses to evaluate the impact of excluding subjects interviewed before job modules were incorporated into the interview (39% of subjects), including in the unexposed group those subjects who were never employed or had only unknown occupations, and the potential effects of 5- and 15-year exposure latency periods. The exposure metrics were adjusted to reflect these latency periods by excluding any work performed within 5 or 15 years of the interview reference date.

## Results

Cases were slightly younger than controls, less likely to be African American, and slightly less likely to have started their first job before 1960, but otherwise were comparable with respect to their distributions by sex, SEER site, and education level (Table 2). This was also true for the subset of subjects assigned as unexposed or probably exposed (i.e., the subset of participants for whom we calculated exposure metrics). The distributions of NHL histologic subtypes among the cases in the overall study sample and the subsample with TCE exposure metrics were also comparable.

We estimated 52 jobs among 45 cases (4% of all cases) and 32 jobs among 27 controls (3% of all controls) to involve probable exposure to TCE. We classified most (*n* = 46, 64%) of the 72 subjects with probable TCE exposure as such because they performed degreasing. Forty-one of these 46 subjects had been administered a job module asking about degreasing; of these subjects, 16 (39%) reported heated degreasing during a time period when TCE was a common vapor degreaser. The remaining five subjects assessed as having probable TCE exposure from degreasing said that they degreased with TCE in the occupational history portion of the interview.

The most common occupation categories among the 84 probably exposed jobs were mechanics and repairers (SOC 61; *n* = 21, 25% of exposed jobs); textile, apparel, and furnishings machine operators (SOC 765; *n* = 7, 8%); assemblers (SOC 772; *n* = 6, 8%); general managers and other top executives (SOC 121, consisting mainly of owners of small businesses; *n* = 6, 7%); and precision metal workers (SOC 681; *n* = 4, 5%). The most common industry categories of the 84 jobs were laundry, cleaning, and garment services (SIC 721; *n* = 10, 12%); tires and inner tubes (SIC 301; *n* = 8, 10%); aircraft and parts (SIC 372; *n* = 6, 7%); motor vehicles and motor vehicle equipment (SIC 371; *n* = 4, 5%); and national security (SIC 971; *n* = 4, 5%). The distribution of exposed jobs classified as mechanics and repairers differed significantly between cases and controls, with 18 of the 21 jobs reported by cases (*n* = 15) and the remaining three jobs reported by two controls (Pearson 1-df χ^2^ = 6.29, *p* = 0.01). Otherwise, the distributions of occupational and industrial groupings did not vary meaningfully between the two groups.

Table 1 summarizes ORs describing the associations between different measures of TCE exposure and NHL. Cases and controls did not differ with respect to the frequency of having an occupational history that involved possible TCE exposure (OR = 1.1 vs. unexposed; 95% CI, 0.9–1.3), but we estimated a statistically nonsignificant higher proportion of cases than controls to have had a work history involving probable exposure to TCE (OR = 1.4; 95% CI, 0.8–2.4). In the analyses of the TCE exposure metrics among probably exposed subjects relative to unexposed subjects, we found that participants who had an average weekly exposure > 150 estimated ppm–hr/week was associated with NHL (OR = 2.5; 95% CI, 1.1–6.1). When we further subdivided this exposure category using the intracategory median defined by the controls’ distribution, the association with NHL for the highest level of average weekly exposure (> 360 estimated ppm–hr/week) became stronger (OR = 7.9; 95% CI, 1.8–34.3). Eight of the 22 subjects with average weekly exposure > 360 estimated ppm–hr/week had a job under the SOC category of mechanics and repairers. When we excluded subjects with such jobs from the analysis, however, the association remained (OR = 4.9; 95% CI, 1.1–22.1). We also observed an association with NHL for cumulative exposure > 112,320 estimated ppm–hours (OR = 2.3; 95% CI, 1.0–5.0), with a stronger association for cumulative exposure above the intracategory median after subdividing the highest exposed category (for > 234,000 estimated ppm–hr: OR = 3.3; 95% CI, 1.1–10.1). The trend test was statistically significant for average weekly exposure (*p* = 0.02) and approached statistical significance for cumulative exposure (*p* = 0.08). However, the associations with categories of average weekly exposure were not suggestive of a monotonic relationship; the ORs were 1.6, 0.5, and 2.5, respectively, for tertiles 1, 2, and 3 (and 0.4 and 7.9 for the tertile 3 below- and above-median subcategories). We observed a similar pattern of associations for categories of cumulative exposure (ORs = 1.4, 0.6, and 2.3, respectively, for tertiles 1, 2, and 3; ORs = 1.4 and 3.3 for tertile 3 below- and above-median categories). Overall, neither duration nor intensity of exposure was associated with NHL, although we observed an association with the lowest tertile of exposure duration (1–6 years of exposure; OR = 2.1; 95% CI, 1.0–4.7).

We observed similar associations with high exposure to TCE within strata defined by sex and age group and upon restriction to non-Hispanic Caucasians (data not shown), and in analyses incorporating latency periods of 5 years (e.g., for average weekly exposure > 150 estimated ppm–hr/week: OR = 2.5; 95% CI, 1.1–6.1; *p*-value for trend = 0.02) and 15 years (OR = 2.3; 95% CI, 1.0–5.8; *p*-value for trend = 0.03). Similarly, an association with high TCE exposure remained when we included in the analysis (and assumed to be unexposed) the 132 cases and 75 controls previously excluded because they were never employed or had only unknown occupations (e.g., for average weekly exposure > 150 estimated ppm–hr/week: OR = 2.3; 95% CI, 1.0–5.4; *p*-value for trend = 0.02). Our findings also did not change when we excluded subjects interviewed before the incorporation of the job modules into the interview [see Supplemental Material, Table 1 (doi:10.1289/ehp.1002106)].

Table 3 summarizes findings from analyses of common histologic subtypes of NHL. Evidence of an association with TCE exposure was strongest for follicular lymphoma (e.g., for average weekly exposure > 150 estimated ppm–hr/week: OR = 3.7; 95% CI, 1.2–11.7; *p*-value for trend = 0.005), although the numbers of exposed cases for each subtype were small (e.g., cases with average weekly exposure > 150 estimated ppm–hr/week: diffuse large B-cell lymphoma, 9; follicular lymphoma, 6; small lymphocytic lymphoma and chronic lymphocytic leukemia, 4).

## Discussion

The results of this case–control study suggest that a high level of exposure to TCE in the workplace is associated with NHL. We observed statistically significant associations with NHL and the highest tertiles of estimated average weekly exposure and cumulative exposure to TCE, although ORs for lower levels of exposure did not suggest monotonic trends for either metric.

Several previous cohort and case–control studies have investigated the association between TCE exposure and NHL risk. The findings from epidemiologic studies published through 2005 have been summarized in three literature reviews (Mandel et al. 2006; Scott and Chiu 2006; Wartenberg et al. 2000). The totality of the evidence from these earlier studies is inconsistent, although findings from the studies that collected more detailed information on TCE-related tasks offer limited support for an association with increased NHL risk. Three small cohort studies of factory workers involving measurements of urinary trichloroacetic acid, the most direct measure of exposure to TCE, observed elevated standardized incidence ratios (SIRs) or standardized mortality ratios (SMRs) for NHL, although the CIs were wide and included unity (Anttila et al. 1995; Axelson et al. 1994; Hansen et al. 2001). Statistically nonsignificant elevated SIRs or SMRs were also observed in three of five occupational cohorts using various combinations of industrial hygienist evaluations, walk-throughs, interviews with employees, monitoring data, and work histories for exposure assessment (Blair et al. 1998; Boice et al. 1999; Morgan et al. 1998; Raaschou-Nielsen et al. 2003; Zhao et al. 2005). Findings from early case–control investigations were inconsistent, with associations reported in some (Hardell et al. 1981, 1994; Persson et al. 1989) but not in others (Greenland et al. 1994; Siemiatycki 1991). Since 2005, three population-based case–control studies of NHL have investigated associations with TCE and chlorinated solvents. Two studies used expert assessments of work history and job module data to evaluate exposure (Fritschi et al. 2005; Seidler et al. 2007); the third study employed a job-exposure matrix (Wang et al. 2009). Borderline statistically significant associations with NHL were observed by Seidler et al. (2007) for the highest level of assessed TCE exposure versus no exposure, and by Wang et al. (2009) for work histories assessed as having a medium or high intensity of TCE exposure versus work histories assessed to have no exposure. The study by Fritschi et al. (2005) showed weak evidence of association with “substantial” levels of exposure to chlorinated solvents versus no exposure.

It has been speculated that possible etiologic heterogeneity across subtypes of NHL, a broad classification for a variety of histologically and clinically distinct malignancies, may have contributed to the inconsistency in published findings regarding TCE (Scott and Chiu 2006). In our study, we observed a stronger association with high TCE exposure for follicular lymphoma. Interestingly, a recent German case–control study also reported stronger TCE associations with this subtype than with other subtypes (Seidler et al. 2007). However, another case–control study conducted among women in Connecticut did not show an association between exposure to any chlorinated solvent and follicular lymphoma (Wang et al. 2009). The subtype-specific findings from all three studies are based on small numbers and should be interpreted with caution. Larger studies are needed to better investigate subtype-specific associations with TCE.

An important strength of this study is the detailed information available on TCE-related tasks. The collected occupational data included a general work history and job- and industry-specific interview modules administered to elicit specific information regarding solvent use. After an extensive literature review (Bakke et al. 2007), we developed task-, job-, industry-, and decade-specific exposure matrices and assessment rules a priori to maximize intrarater reliability. All of these data were considered by an expert industrial hygienist when assigning several parameters for potential exposure to TCE. This approach enabled the calculation of exposure metrics restricted to subjects rated as having probable TCE exposure. The benefit of using an improved exposure assessment may be reflected in the pattern of findings that have emerged from the three analyses involving occupational exposure to solvents conducted within the NCI-SEER study to date. An initial analysis based solely upon occupational history data did not suggest a clear association with NHL for occupations or industries that can involve exposure to TCE and other chlorinated solvents (Schenk et al. 2009), whereas a subsequent NCI-SEER analysis using limited data collected from the interview modules suggested a possible association with a high frequency of performing degreasing tasks (Purdue et al. 2009), and in the present analysis we observed a statistically significant association with high estimated exposure levels to TCE. Another strength of this population-based study was its large sample size, which enabled the identification of a small number of individuals highly exposed to TCE (a small subgroup of the general population) and permitted an opportunity to explore whether associations varied by histologic subtype.

In spite of its large size, an important limitation of this study is the small number of subjects estimated to be highly exposed to TCE. As a consequence, we cannot rule out the possibility that our findings, and the subtype-specific results in particular, may have arisen because of chance. We also cannot rule out selection bias as an explanation for our findings, because the participation rate among controls was comparatively low, although we previously estimated demographic and socioeconomic differences between control participants and nonparticipants to be generally minor (Shen et al. 2008). Also, some relevant jobs did not trigger modules. The overwhelming cause of this was the timing of the incorporation of the modules into the interview. It seems unlikely that the lack of modules could account for our findings, because it is improbable that module data missingness differed between controls and cases in an exposure-dependent manner. Moreover, a sensitivity analysis excluding subjects interviewed before the incorporation of the job modules into the interviews yielded virtually identical findings [see Supplemental Material, Table 1 (doi:10.1289/ehp.1002106)]. Interviewer bias is implausible given the highly structured, controlled nature of the interview as administered using the CAPI; however, as with other case–control studies, we cannot rule out the possibility that bias due to differential recall of occupational tasks and their characteristics by cases and controls may have been introduced into the study. In addition, similar to most population-based case–control studies of occupational exposures, we were not able to validate our exposure estimates. Evaluations of expert-based exposure assessment methods in case–control studies have been inconsistent, with widely varying levels of validity reported (Teschke et al. 2002). Some evaluations suggest that expert-based methods yield parts per million estimates that are consistently higher than actual measurements (Cherrie and Hughson 2005; Cherrie and Schneider 1999). Given this absence of validation data for our assessments, and that the estimates of exposure intensity are not based on direct monitoring of the subjects’ work environment, the parts per million estimates should be interpreted with caution, and their absolute values should not be used for risk assessment.

Lastly, a limitation of our study is the lack of assessment data on other widely used chlorinated solvents. Whereas TCE use in vapor degreasing was extensive from the early 1920s through the early 1970s, environmental and health concerns have since led to a decline in its use, with other chlorinated solvents (methylene chloride, tetrachloroethylene, 1,1,1-trichloroethane) increasingly used instead (Bakke et al. 2007). There is limited epidemiologic evidence suggesting associations between these and other chlorinated solvents (e.g., carbon tetrachloride) and NHL (Ruder 2006; Seidler et al. 2007; Wang et al. 2009). Although our exposure assessment took into account the secular trends in TCE use, we nonetheless cannot rule out the possibility that our findings were confounded by effects of other chlorinated solvents. Other nonagricultural occupational exposures, benzene in particular, have been studied as possible risk factors for NHL, with equivocal results (Hartge et al. 2006). It is unlikely that other occupational agents had an association with NHL of sufficiently large size, and were correlated with TCE exposure strongly enough, to have materially confounded our findings (Blair et al. 2007).

## Conclusions

In this large U.S. population-based case– control study, we observed an association with NHL for high levels of estimated occupational exposure to TCE. By structured probing of study participants’ jobs that might have involved TCE use, and having the resulting detailed interview data reviewed in concert with the exposure literature by an industrial hygienist to estimate quantitative exposures, we observed statistically significant elevated ORs with high exposure that were not otherwise detected using less detailed exposure assessment methods. These findings further support the existing epidemiologic evidence suggesting that TCE is a lymphomagen, with the caveat that we were unable to adjust for exposure to other chlorinated solvents. Additional investigation of the association between TCE exposure and NHL, both overall and by histologic subtype, is warranted.

## Figures and Tables

**Table 1:** Analysis of estimated occupational exposure to TCE and NHL within the NCI-SEER study, 1998–2001.

Exposure metric	Exposure level	Controls [*n* (%)]	Cases [*n* (%)]	OR^a^ (95% CI)	*p*-Value for trend
Any exposure to TCE	Unexposed	539 (54.9)	599 (50.4)	1.0	
	Possible^b^	416 (42.4)	545 (45.8)	1.1 (0.9–1.3)	
	Probable^c^	27 (2.8)	45 (3.8)	1.4 (0.8–2.4)	

Average weekly exposure^d^ (estimated ppm–hr per week)	0	539 (95.2)	599 (93.0)	1.0	
	1–60	9 (1.6)	15 (2.3)	1.6 (0.7–3.8)	
	61–150	11 (1.9)	7 (1.1)	0.5 (0.2–1.4)	
	> 150	7 (1.2)	23 (3.6)	2.5 (1.1–6.1)	
	151–360^e^	5 (0.9)	3 (0.5)	0.4 (0.1–1.8)	
	> 360^e^	2 (0.4)	20 (3.1)	7.9 (1.8–34.3)	
	per 90 estimated ppm–hr/week^f^			1.11 (1.02–1.21)	0.02

Years exposed^d^	0	539 (95.2)	599 (93.0)	1.0	
	1–6	9 (1.6)	22 (3.4)	2.1 (1.0–4.7)	
	7–16	9 (1.6)	10 (1.6)	0.8 (0.3–2.1)	
	> 16	9 (1.6)	13 (2.0)	1.3 (0.5–3.1)	
	17–24^e^	5 (0.9)	6 (0.9)	1.0 (0.3–3.4)	
	> 24^e^	4 (0.7)	7 (1.1)	1.7 (0.5–5.8)	
	per 10 years^f^			1.13 (0.85–1.51)	0.40

Cumulative exposure^d^ (estimated ppm–hr)	0	539 (95.2)	599 (93.0)	1.0	
	1–46,800	9 (1.6)	14 (2.2)	1.4 (0.6–3.3)	
	46,801–112,320	9 (1.6)	7 (1.1)	0.6 (0.2–1.7)	
	> 112,320	9 (1.6)	24 (3.7)	2.3 (1.0–5.0)	
	112,321–234,000^e^	5 (0.9)	8 (1.2)	1.4 (0.5–4.4)	
	> 234,000^e^	4 (0.7)	16 (2.5)	3.3 (1.1–10.1)	
	per 65,520 estimated ppm–hr^f^			1.10 (0.99–1.22)	0.08

Average exposure intensity (estimated ppm)^d^	0	539 (95.2)	599 (93.0)	1.0	0.41
	1–99	14 (2.5)	23 (3.6)	1.5 (0.8–2.9)	
	> 99	13 (2.3)	22 (3.4)	1.3 (0.7–2.7)	
	per 99 estimated ppm^f^			1.18 (0.80–1.76)	0.41

a ORs were computed using unconditional logistic regression adjusted for age group, sex, SEER center, race, and education.

b Subjects with one or more jobs with an assigned probability of TCE exposure no higher than < 50%; these subjects were excluded from subsequent analyses of TCE exposure metrics.

c One or more jobs with an assigned probability of TCE exposure of ≥ 50%.

d Includes subjects assessed as unexposed (539 controls, 599 cases) or probably exposed (27 controls, 45 cases).

e Subdivision of highest exposure category using within-category median among controls.

f The selected scale represents the difference between the second and third tertiles among exposed controls.

**Table 2:** Selected characteristics of participants in the NCI-SEER study, 1998–2001.

	All subjects [*n* (%)]		Subjects assessed as unexposed or probably exposed to TCE [*n* (%)]	
Characteristics	Controls (*n* = 982)	Cases (*n* = 1,189)	Controls (*n* = 566)	Cases (*n* = 644)
Age at reference date (years)				

< 35	53 (5.4)	68 (5.7)	44 (7.8)	56 (8.7)
35–44	98 (10.0)	153 (12.9)	51 (9.0)	83 (12.9)
45–54	185 (18.8)	261 (22.0)	100 (17.7)	115 (17.9)
55–64	230 (23.4)	316 (26.6)	136 (24.0)	174 (27.0)
≥ 65	416 (42.4)	391 (32.9)	235 (41.5)	216 (33.5)

Sex				

Female	458 (46.6)	523 (44.0)	238 (42.1)	284 (44.1)
Male	524 (53.4)	666 (56.0)	328 (58.0)	360 (55.9)

Race				

White	787 (80.1)	1,014 (85.3)	452 (79.9)	541 (84.0)
African American	132 (13.4)	91 (7.7)	75 (13.3)	56 (8.7)
Other	63 (6.4)	84 (7.1)	39 (6.9)	47 (7.3)

Study center				

Detroit	144 (14.7)	209 (17.6)	79 (14.0)	109 (16.9)
Iowa	273 (27.8)	352 (29.6)	153 (27.0)	181 (28.1)
Los Angeles	273 (27.8)	310 (26.1)	157 (27.7)	178 (27.6)
Seattle	292 (29.7)	318 (26.8)	177 (31.3)	176 (27.3)

Years of education				

< 12	97 (9.9)	118 (9.9)	46 (8.1)	63 (9.8)
12–15	584 (59.5)	734 (61.7)	335 (59.2)	376 (58.4)
≥ 16	301 (30.7)	336 (28.3)	185 (32.7)	204 (31.7)
Missing	0 (0.0)	1 (0.1)	0 (0.0)	1 (0.1)

Year of first employment				

< 1950	239 (24.3)	247 (20.8)	131 (23.1)	126 (19.6)
1950–1959	274 (27.9)	290 (24.4)	153 (27.0)	161 (25.0)
1960–1969	225 (22.9)	284 (23.9)	118 (20.9)	131 (20.3)
1970–1979	154 (15.7)	233 (19.6)	91 (16.1)	119 (18.5)
≥ 1980	90 (9.1)	135 (11.4)	73 (12.9)	107 (16.6)

NHL histologic type^a^				

Diffuse large B-cell		366 (30.8)		211 (32.8)
Follicular		293 (24.6)		146 (22.7)
Small lymphocytic lymphoma/chronic lymphocytic leukemia		141 (11.9)		79 (12.3)
Other or not otherwise specified		389 (32.7)		208 (32.4)

a Cases only.

**Table 3:** Analysis of estimated occupational exposure to TCE and selected NHL histologic subtypes within the NCI-SEER study, 1998–2001.

Exposure metric	Controls *n*	Diffuse large B-Cell lymphoma		Follicular lymphoma		Small lymphocytic lymphoma/chronic lymphocytic leukemia	
		*n*	OR^a^ (95% CI)	*n*	OR (95% CI)	*n*	OR (95% CI)
Any exposure to TCE							

Unexposed	539	200	1.0	133	1.0	68	1.0
Possible^b^	416	11	0.9 (0.7–1.2)	147	1.4 (1.1–1.9)	62	1.0 (0.7–1.5)
Probable^c^	27	155	0.9 (0.5–2.0)	13	2.1 (1.0–4.2)	11	2.7 (1.2–5.8)

Average weekly exposure (estimated ppm–hr/week)^d^							

0	539	200	1.0	133	1.0	68	1.0
1–150	20	2	0.3 (0.1–1.1)	7	1.9 (0.8–4.8)	7	2.6 (1.0–6.8)
> 150	7	9	2.5 (0.9–7.1)	6	3.7 (1.2–11.7)	4	3.0 (0.8–11.4)
per 90 estimated ppm–hr/week^e^			1.11 (1.01–1.23) *p*_trend_ = 0.03		1.15 (1.04–1.28) *p*_trend_ = 0.005		1.09 (0.96–1.24) *p*_trend_ = 0.16

Cumulative exposure (estimated ppm–hr)^d^							

0	539	200	1.0	133	1.0	68	1.0
1–112,320	18	3	0.4 (0.1–1.4)	7	1.9 (0.8–4.8)	7	2.7 (1.0–7.0)
> 112,320	9	8	1.9 (0.7–5.2)	6	3.3 (1.1–10.0)	4	2.7 (0.8–9.5)
per 65,520 estimated ppm–hr^e^			1.07 (0.94–1.22) *p*_trend_ = 0.29		1.17 (1.04–1.32) *p*_trend_ = 0.01		1.11 (0.96–1.27) *p*_trend_ = 0.16

a ORs computed using polytomous regression adjusted for age group, sex, SEER center, race, and education.

b Subjects with one or more jobs with an assigned probability of TCE exposure no higher than < 50%; these subjects were excluded from subsequent analyses of TCE exposure metrics.

c One or more jobs with an assigned probability of TCE exposure of ≥ 50%.

d Includes subjects assessed as unexposed (539 controls, 599 cases) or probably exposed (27 controls, 45 cases).

e The selected scale represents the difference between the second and third tertiles among exposed controls.
